# Design, Synthesis, and Structural Characterization of Novel Diazaphenothiazines with 1,2,3-Triazole Substituents as Promising Antiproliferative Agents

**DOI:** 10.3390/molecules24234388

**Published:** 2019-11-30

**Authors:** Beata Morak-Młodawska, Krystian Pluta, Małgorzata Latocha, Małgorzata Jeleń, Dariusz Kuśmierz

**Affiliations:** 1 Department of Organic Chemistry, Faculty of Pharmaceutical Sciences, The Medical University of Silesia, Jagiellońska 4, 41-200 Sosnowiec, Poland; pluta@sum.edu.pl (K.P.); manowak@sum.edu.pl (M.J.); 2 Department of Cell Biology, Faculty of Pharmaceutical Sciences, The Medical University of Silesia, Jedności 8, 41-200 Sosnowiec, Poland; mlatocha@sum.edu.pl (M.L.); dkusmierz@sum.edu.pl (D.K.)

**Keywords:** diazaphenothiazines, 1,2,3-triazole ring, antiproliferative activity, intracellular apoptosis pathway, *H3*, *TP53*, *CDKN1A*, *BAX/BCL-2* ratio

## Abstract

A series of novel 1,2,3-triazole-diazphenothiazine hybrids was designed, synthesized, and evaluated for anticancer activity against four selected human tumor cell lines (SNB-19, Caco-2, A549, and MDA-MB231). The majority of the synthesized compounds exhibited significant potent activity against the investigated cell lines. Among them, compounds **1d** and **4c** showed excellent broad spectrum anticancer activity, with IC_50_ values ranging from 0.25 to 4.66 μM and 0.25 to 6.25 μM, respectively. The most promising compound **1d**, possessing low cytotoxicity against normal human fibroblasts NHFF, was used for gene expression analysis using reverse transcription–quantitative real-time PCR (RT–qPCR). The expression of *H3, TP53, CDKN1A, BCL-2,* and *BAX* genes revealed that these compounds inhibited the proliferation in all cells (*H3*) and activated mitochondrial events of apoptosis (*BAX/BCL-2*).

## 1. Introduction

The phenothiazines were the first synthetic agents to be used successfully in psychiatry as neuroleptics. The properties of this group of heterocyclic compounds was discovered in the 1950s and provoked revolutions in contemporary medicine [1]. Currently, they are also well-known clinically as substances with antibacterial, antiviral, anti-inflammatory, and anti-tuberculosis properties. There have also been numerous reports on the antitumor activity of these drugs [2,3,4,5,6]. Neuroleptic phenothiazines became the foundation for numerous studies on modified phenothiazine systems that show a wide spectrum of biological activities, including: anticancer, antibacterial, antifungal, antiviral, and antiparasitic. This rich scientific material is the subject of numerous publications that can be found in the world literature every year [7,8,9,10,11,12,13,14,15,16].

The replacement of two benzene rings with the pyridine rings leads to dipyridothiazines. These diazaphenothiazines were found to be an excellent scaffold for novel anticancer agents with high safety profile. We synthesized dipyridothiazines of the 1,6-, 1,8-, 2,7-, and 3,6-diazaphenothiazine structures with varied alkyl, aryl, heteroaryl, dialkylaminoalkyl, amidoalkyl, sulfonamidoalkyl, and dialkylaminoalkynyl substituents at the thiazine nitrogen atom. Some selected compounds showed very promising anticancer, immunosuppressant, and antioxidant activities, and low toxicity [17,18,19,20,21,22,23,24,25].

The 1,2,3-triazoles, due to their unique chemical and structural properties, have received much attention over the past decades, and have been well-recognized for their broad range of pharmacological properties. This heterocycle has been well exploited for the generation of many medicinal scaffords exhibiting anti-HIV, anticancer, antimicrobial, and antidepressant activities [26,27,28].

Several triazoles (e.g., cefatrizine, tazobactum) have been used in medicine as β-lactam antibiotics [29]. Additionally 1,2,3-triazoles can form hydrogen bonds, which play important roles for bioavailability and solubility [30]. The triazole ring is the biological linker and exhibits bioisosteric effects on various heteroaromatic and aromatic rings [31,32,33].

The association of the potency of dipyridothiazine and 1,2,3-triazole pharmacophores under the same molecular frame was the target of our on-going idea for rational design of new biologically active compounds. The bioactive dipyridothiazines (1,6-, 1,8-, 2,7-, and 3,6-diazaphenothiazine **1-4**) were combined with the 1,2,3-triazole system, using 1,3-dipolar cycloaddition reaction between 2-propynyl derivatives of dipyridothiazines **(1**–**4)a** and some selected organic azides.

For the triazole hybrids, the anticancer action on the selected cancer cell lines: glioblastoma SNB-19, colorectal carcinoma Caco-2, lung cancer A549, and breast cancer MDA-MB231 were investigated. The cytotoxicity was determined towards normal human fibroblasts NHDF. In order to understand the mechanism of action and the effects on cancer biology, the expression of *H3, TP53, CDKN1A, BCL-2* and *BAX* genes was detected for the most active compound by the RT-qPCR method.

## 2. Results

### 2.1. Chemistry

**Scheme 1:**
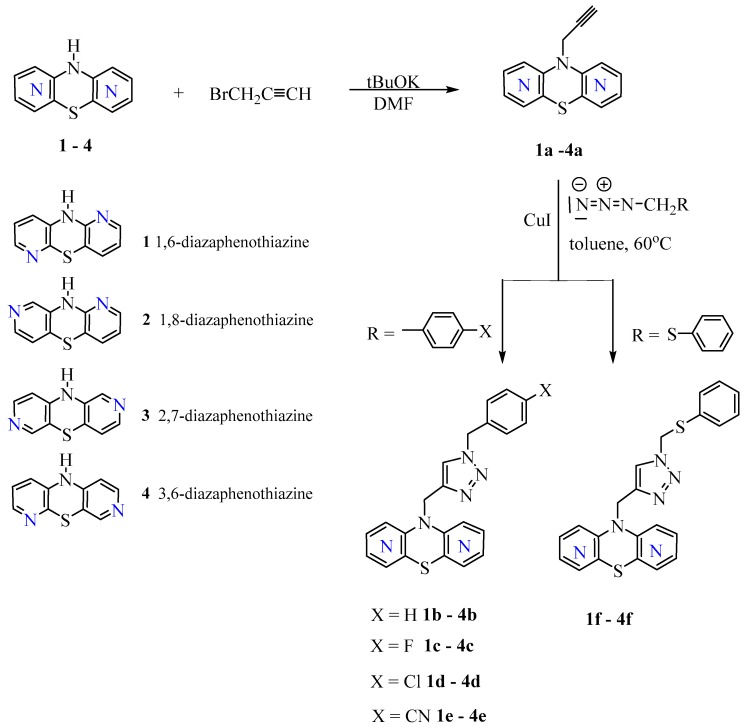
Synthesis of 1,2,3-triazole derivatives of dipyridothiazines (**1**–**4)b**–**f.**

The synthetic strategy applied for the preparation of the new triazole hybrids of diazphenothiazines was founded on selection of dipyridothiazines **1**–**4** as the building block laid open for assembling the 1,2,3-triazole ring, as presented in Scheme 1.

The starting materials: 10H-1,6-, 1,8-, 2,7-, and 3,6-diazaphenothiazines **1**–**4**, were transformed with 2-propynyl bromide into the propynyl derivatives **(1**–**4)a** according to the described synthesis [20,21,22,23], and further using “click chemistry”-1,3-dipolar cycloadditon (with selected azides, in toluene, in the presence of CuI as a catalyst) into substituted triazole derivatives of diazaphenothiazines (**1**–**4)b**–**f** in good yields (67–82%).

Bearing in mind the fact of significant biological activities of triazole systems with various benzylic and phenyl substituents [34,35,36], the following selected azides (benzyl azide, p-fluorobenzyl azide, *p*-chlorobenzyl azide, *p*-cyanobenzyl azide, and phenylthiometyl azide) were selected for the 1,3-dipolar cycloaddition.

The identification of the product structure was based on ^1^H, ^13^C NMR spectra, 2D NMR experiments: COSY and ROESY, and mass spectrometry HRMS.

The crude products of the reactions were separated by column chromatography to obtain pure final derivatives (**1**–**4)b**–**f** with good yields.

**Figure 1:**
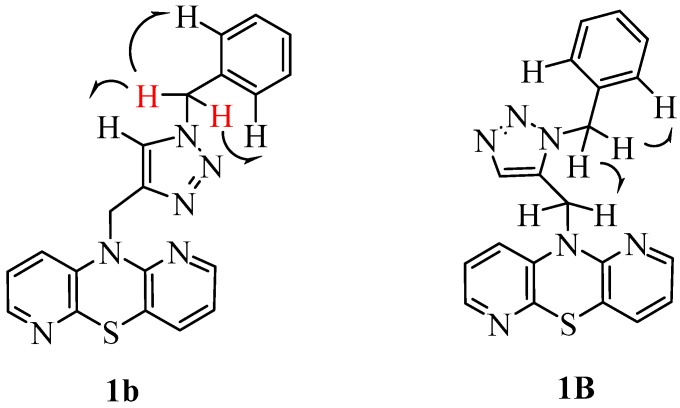
The ROESY experiment for triazole derivative **1b**.

On the basis of previous studies, the reaction of 2-propynyl derivatives with organic azides can led to 1,4- and/or 1,5-regioisomers [26,28]. Bearing in mind the literature, using a CuI catalyst, a 1,4 regioisomer is obtained selectively [28,34]. Nevertheless, the structure of the products received had to be unambiguously confirmed. For this reason, the 2D NMR ROESY experiment of compound **1b** was carried out (Figure 1, Table 1, Supplementary Material). For triazole hybrid **1b**, protons of the benzylic group (CH_2_N_Tr_ where Tr—triazole ring) intercorrelated with aromatic proton of the 1,2,3-triazole ring and *orto*-protons of the benzene ring. However, if a substitution product of 1,5-regioisomer **1B** was formed in the reaction, intercorrelation between the CH_2_ group at the thiazine nitrogen atom (CH_2_N_Th_ where Th—thiazine ring) with the CH_2_ group at the triazole ring (CH_2_N_Tr_) would be expected but was not observed. 

This we consider to be proof of the presence of 1,4-disubstituted 1,2,3-triazole ring. The full correlation between the neighbouring protones showed COSY spectra (Table 1, Supplementary Material).

### 2.2. Anticancer Activity

Encouraged by our previous promising results in the field of antiproliferative activity of dipyridothiazines [17,18,19,20,21,22,23,24,25] as well as by the good anticancer activity of the 1,2,3-triazoles [26,27,28,29,30,31,32,33], we decided to combine the biological potentials of dipyridothiazines and triazole moieties, hoping to obtain compounds with better activity. The antiproliferative activity of all 20 new 10-substituted 1,2,3-triazole derivatives of dipyridothiazines was tested in vitro against cancer cell lines: glioblastoma SNB-19, colorectal carcinoma Caco-2, lung cancer A549, and breast cancer MDA-MB231 using the WST-1 assay. Normal human fibroblasts NHDF were used as a control. Cisplatin was used as a positive control to induce cell death. The results of the cytotoxicity studies were summarized in Table 2.

The most active derivative in relation to all tumor groups was compound **1d**, which structure contained the *p*-chlorobenzyl substituent (IC_50_ = 0.25–4.66 μM). This compound also showed relatively low cytotoxicity in relation to normal fibroblasts NHDF due to the vacancy of the examined controls. This compound showed higher activity compared to cisplatin (IC_50_ = 0.60–10.53 μM). In the 1,6-diazaphenothiazine group, derivative **1c** with the *p*-fluorobenzyl substituent was also characterized by high anticancer activity in relation to SNB-19 and MDA-MB231 cell lines (IC_50_ = 5.72–7.82 μM). 1,8-Diazaphenothiazine **2d** (with the *p*-chlorobenzyl substituent in the 1,2,3-tiazole system) showed high cytotoxic activity in relation to lung cancer cell lines A-549 (IC_50_ = 1.82 μM). This compound was also active against Caco-2 (IC_50_ = 11.42 μM) and MDA-MB231 (IC_50_ = 4.71 μM) cell lines. In the group of 1,8-diazaphenothiazines derivative **2e** with the *p*-cyanobenzyl substituent stood out. The compound showed significant activity against glioblastoma SNB-19 (IC_50_ = 10.62 μM) and breast cancer MDA-MB231 (IC_50_ = 12.04 μM). 2,7-Diazaphenothiazine **3b** (with the benzyl substituent in 1,2,3-tiazole system) showed high activity against all investigated cancer cell lines (IC_50_ = 0.26–2.07 μM). On the other hand derivative **3f** (with the phenyltiomethyl substituent in 1,2,3-triazole system) acted selectively towards A549 and MDA-MB231 cell lines. In the 3,6-diazaphenothiazine group, the highest anticancer activity was characterized by **4c** derivative with the *p*-fluorobenzyl substituent (IC_50_ = 0.25–6.25 μM). This compound showed high cytotoxicity in relation to all cancer cells lines, however, it also showed high cytotoxicity in relation to normal human fibroblasts (IC_50_ = 2.85 μM).

Most of the tested compounds (**1**–**4)b**–**f** were characterized by significant antiproliferative activity in relation to the examined cancer cell lines and relatively weak cytotoxicity with respect to normal human fibroblasts. This activity was largely dependent on the location of nitrogen atoms in isomeric dipyridothiazines as well as on the substituent in the benzene ring of the 1,2,3-tiazole system.

### 2.3. Apoptosis Assay

10-((1-(4-Chlorobenzyl)-1H-1,2,3-triazol-4-yl)methyl)-1,6-diazaphenothiazine (**1d**)—the most active compound—was selected to study the mechanism of anticancer activity using RT-qPCR. In this method the gene transcriptional activities of proliferation marker (*H3*), cell cycle regulator (*TP53* and *CDKNIA*), and intracellular apoptosis pathway (*BCL-2* and *BAX*) were analyzed. The results obtained on four cancer cell lines are collected in Table 3. It has been known that the growth, metabolism, and eventual death of the cells in the body are controlled by hundreds of genes working together [35,36,37]. The gene encoding histone *H3* plays a crucial role in regulation of the expression of the genetic information included in the DNA. Compound **1d** strongly changed the mRNA copy number of the histone *H3* gene in all cancer lines which has an influence on the modification of the chromatin structure in the cells.

It is well known, that cellular stresses such as the DNA damage and oncogene activation can change the expression of the *TP53* gene encoding the p53 protein which is called the guardian of the genome of the cell. The p53 protein influences cell cycle arrest by changing the expression of *CDKN1A* gene encoding the p21 protein. The essential function of the p21 protein is to arrest cell cycle progression by inhibiting the activity of cyclin-dependent kinases [37,38,39].

Compound **1d** significantly interfered with the amount of mRNA copies of *TP53* in all investigated cancer lines. There was also a strong decline of *CDKN1A* copies in all cancer cells what suggests the possibility of participation in cell cycle arrest.

In biochemical processes of cells, the p53 protein determines the standard balance between the expression of the proapoptotic *BAX* gene and the antiapoptotic *BCL-2* gene. The balance between these proteins determines whether the cell is undergoing apoptosis or interrupting the process. It is believed that the main mechanism of action of the *BCL-2* family of proteins regulates the release of cytochrome c from mitochondria by changing the mitochondrial membrane permeability [40,41].

Compound **1d** remarkably reduced the expression of *BCL-2* in SNB-19, A549 and MDA-MB231 cancer lines, but there was a slight increase in Caco-2 cell line. On the other hand, a decrease of the expression of *BAX* was observed in all investigated cancer cell lines.

Analysis of the gene expression ratio *BCL-2/BAX* in MDA-MB231 cell showed activation of the mitochondrial apoptosis as the internal pathway of cell death. Transcriptional activity of these genes in the SNB-19, Caco-2, and A549 cells suggests a different way of cell death, possibly associated with the external pathway of apoptosis. The presented results indicate the possibility of a two-way mechanism of apoptosis caused by the hybrid of diazaphenothiazine with the 1,2,3-triazole system. However, further research is required to fully confirm this hypothesis.

## 3. Materials and Methods

### 3.1. Chemistry

The standard NMR spectra were recorded on Bruker Avance spectrometers (^1^H at 600 MHz, ^13^C at 150 MHz) in CDCl_3_. Two-dimensional COSY and ROESY, spectra of selected compounds were recorded on a Bruker Avance spectrometer at 600 MHz. The HRMS spectra (EI—electro impact ionisation) were run on a Brucker Impact II. The thin layer chromatography was performed on aluminium oxide 60 F_254_ neutral (type E) (Merck 1.05581) with CHCl_3_-EtOH (10:1 v/v) as eluent.

*10H-1,6-diazaphenothiazine* (**1**), *10H-1,8-diazaphenothiazine* (**2**), *10H-2,7-diazaphenothiazine* (**3**) and *10H-3,6-diazaphenothiazine* (**4**) were obtained and were transformed into 10-propynyl derivatives (**1**–**4)a** according to previously described methods [18,20,21,22,23].

General Procedure for Synthesis of Compounds (**1**–**4**)**b.** To a solution of 10-propynyl diazaphenothiazine (**1**–**4)a** (0.120 mg, 0.5 mmol) and copper iodide (I) (catalytic amount) in dry toluene (5 mL), a corresponding organic azide (0.510 mmol) was added. The reaction mixture was stirred and heated at 70 °C for 48 h. Then the solvent mixture was distilled under reduced pressure. The dry residue was dissolved in CHCl_3_ and purified by column chromatography (aluminum oxide 90 active neutral, Merck 1.01077.2000, CHCl_3_ as eluent) to give pure triazole derivatives **1b**–**4f**:

*10-[(1-Benzyl-1H-1,2,3-triazol-4-yl)-methyl]-1,6-diazaphenothiazine* (**1b**). (136 mg, 76%) brown oil. ^1^H NMR (CDCl_3_) δ: 5.22 (s, 2H, CH_2_N_Th_), 5.49 (s, 2H, CH_2_N_Tr_), 6.76 (dd, *J =* 7.2 Hz, *J =* 4.8 Hz 1H, H_3_), 6.93 (dd, *J =* 7.2 Hz, *J =* 4.8 Hz, 1H, H_8_), 7.23 (m, 3H, 2H_Ph_, 1H_4_), 7.35 (m, 3H, 3H_Ph_), 7.46 (d, *J =* 7.2 Hz, 1H, H_9_), 7.61 (s, 1H, H_Tr_), 7.92 (m, 2H, H_7_ + H_2_). ^13^C NMR: 41.94, 54.13, 116.53, 118.39, 121.57, 122.21, 124.06, 127.87, 128.68, 129.07, 134.46, 134.65, 138.52, 142.69, 143.89, 144.82, 144.88, 152.12. HRMS (EI) *m*/*z* for: [C_20_H_16_N_6_S + H] calc. 373.1235 Found: 373.1225

*10-[(1-(4-Fluorobenzyl)-1H-1,2,3-triazol-4-yl)methyl]-1,6-diazaphenothiazine* (**1c**). (140 mg, 74%) yellow oil. ^1^H NMR (CDCl_3_) δ: 5.22 (s, 2H, CH_2_N_Th_), 5.46 (s, 2H, CH_2_N_Tr_), 6.77 (m, 1H, H_3_), 6.93 (m, 1H, H_8_), 7.05 (m, 2H, 2H_Ph_), 7.23 (m, 3H, 2H_Ph_, 1H_4_), 7.45 (m, 1H, 1H_9_), 7.59 (s, 1H, H_Tr_), 7.94 (m, 2H, H_7_ + H_2_). ^13^C NMR: 41.94, 53.40, 115.73, 116.01, 116.53, 118.40, 121.49, 122.17, 123.91, 127.78, 129.83, 130.00, 130.05, 134.46, 138.47, 142.79, 143.93, 144.79, 145.03, 152.12. HRMS (EI) *m*/*z* for: [C_20_H_15_FN_6_S + H] calc. 391.1141 Found: 391.1145.

*10-[(1-(4-Chlorobenzyl)-1H-1,2,3-triazol-4-yl)methyl]-1,6-diazaphenothiazine* (**1d**). (139 mg, 70%). brown oil. ^1^H NMR (CDCl_3_) δ: 5.21 (s, 2H, CH_2_N_Th_), 5,46 (s, 2H, CH_2_N_Tr_), 6.77 (m, 1H, H_3_), 6.92 (m, 1H, H_8_), 7.18 (m, 2H, 2H_Ph_), 7.23 (m, 1H, H_4_), 7.32 (m, 2H, 2H_Ph_), 7.44 (m, 1H, H_9_), 7.61 (s, 1H, 1H_Tr_), 7.94 (m, 2H, H_7_ + H_2_). ^13^C NMR: 41.91, 54.40, 116.61, 118.43, 121.46, 122.19, 124.02, 129.25, 129.28, 133.13, 134.47, 134.72, 138.46, 142.80, 143.92, 144.82, 145.10, 152.09. HRMS (EI) *m*/*z* for: [C_20_H_15_ClN_6_S + H] calc. 407.0846 Found: 407.0836

*10-[(1-(4-Cyanobenzyl)-1H-1,2,3-triazol-4-yl)methyl]-1,6-diazaphenothiazine* (**1e**). (130 mg, 67%) brown oil. ^1^H NMR (CDCl_3_) δ: 5.22 (s, 2H, CH_2_N_Th_), 5.55 (s, 2H, CH_2_N_Tr_), 6.76 (m, 1H, H_3_), 6.91 (m, 1H, H_8_), 7.21 (m, 1H, H_4_), 7.28 (m, 2H, 2H_Ph_), 7.41 (m, 1H, H_9_), 7.63 (m, 2H, 2H_Ph_), 7.68 (s, 1H, H_Tr_), 7.93 (m, 2H, H_7_ + H_2_). ^13^C NMR: 41.85, 53.36, 112.63, 116.60, 118.41, 120.29, 121.37, 122.18, 124.43, 128.29, 129.07, 132.84, 134.50, 138.41, 142.82, 143.92, 144.81, 145.32, 152.00. HRMS (EI) *m*/*z* for: [C_21_H_15_N_7_S + H] calc. 398.1189 Found: 398.1176

*10-[(1-Phenylthiomethyl-1H-1,2,3-triazolo-4-ylo)methyl*]-*1,6-diazaphenothiazine* (**1f**). (150 mg, 75%) brown oil. ^1^H NMR (CDCl_3_) δ: 5.18 (s, 2H, CH_2_N_Th_), 5.55 (s, 2H, CH_2_N_Tr_), 6.79 (m, 1H, H_3_), 6.92 (m, 1H, H_8_), 7.22 (m, 6H, 5H_Ph_, H_9_), 7.34 (m, 1H, H_4_), 7.49 (s, 1H, 1H_Tr_), 7.92 (m, 2H, H_7_ + H_2_). ^13^C NMR: 41.77, 54.05, 116.49, 118.43, 121.38, 122.17, 123.70, 128.88, 129.36, 131.51, 132.89, 134.48, 138.43, 142.76, 143.91, 144.87, 144.91, 152.01. HRMS (EI) *m*/*z* for: [C_20_H_16_N_6_S_2_ + H] calc. 405.0956 Found: 405.0957

*10-[(1-Benzyl-1H-1,2,3-triazol-4-yl)-methyl]-1,8-diazaphenothiazine* (**2b**). (135 mg, 75%) brown oil. ^1^H NMR (CDCl_3_) δ: 5.31 (s, 2H, CH_2_N_Th_), 5.50 (s, 2H, CH_2_N_Tr_), 6.78 (m, 1H, H_3_), 6.93 (m, 1H, H_6_), 7.19 (m, 1H, H_4_), 7.20 (m, 2H, H_Ph_), 7.35 (m, 3H, H_Ph_), 7.51 (s, 1H, H_Tr_), 7.96 (m, 1H, H_2_), 8.04 (m, 1H, H_7_), 8.29 (m, 1H, H_9_). ^13^C NMR: 41.76, 54.13, 114.45, 118.80, 120.76, 123.35, 126.57, 127.90, 128.65, 129.08, 129.22, 134.17, 134.41, 134.68, 142.40, 144.31, 145.59, 152.65. HRMS (EI) *m*/*z* for: [C_20_H_16_N_6_S + H] calc. 373.1235 Found 373.1235. 

*10-[(1-(4-Fluorobenzyl)-1H-1,2,3-triazol-4-yl)methyl]-1,8-diazaphenothiazine* (**2c**). (142 mg, 76%) yellow oil. ^1^H NMR (CDCl_3_) δ:5.32 (s, 2H, CH_2_N_Th_), 5.46 (s, 2H, CH_2_N_Tr_), 6.78 (m, 1H, H_3_), 6.79 (m, 1H, H_6_), 7.05 (m, 2H, 2H_Ph_), 7.19 (m, 1H, H_4_), 7.22 (m, 2H, 2H_Ph_), 7.48 (s, 1H, H_Tr_), 7.96 (m, 1H, H_2_), 8,08 (m, 1H, H_7_), 8,28 (m, 1H, H_9_). ^13^C NMR: 41.83, 53.35, 114.89, 115.73, 115.87, 115.98, 118.59, 123.22, 129.73, 129.78, 130.00, 130.55, 130.57, 131.60, 134.36, 135.79, 143.81, 144.87, 145.39, 153.00. HRMS (EI) *m*/*z* for: [C_20_H_15_FN_6_S + H] calc. 391.1141 Found: 391.1143.

*10-[(1-(4-Chlorobenzyl)-1H-1,2,3-triazol-4-yl)methyl]-1,8-diazaphenothiazine* (**2d**). 140 mg, 71%). brown oil. ^1^H NMR (CDCl_3_) δ: 5.31 (s, 2H, CH_2_N_Th_), 5.47 (s, 2H. CH_2_N_Tr_), 6.81 (m, 1H, H_3_), 6.93 (m, 1H, H_6_), 7.18 (m, 2H, 2H_Ph_), 7.21 (m, 1H, H_4_), 7.34 (m, 2H, 2H_Ph_), 7.51 (s, 1H, H_Tr_), 7.97 (m, 1H, H_2_), 8.04 (m, 1H, H_7_), 8.29 (m, 1H, H_9_). ^13^C NMR: 41.86, 53.25, 114.49, 115.72, 115.67, 115.98, 118.61, 123.20, 129.74, 129.76, 130.00, 130.59, 130.47, 131.66, 134.16, 135.69, 143.81, 144.77, 145.49, 153.02. HRMS (EI) *m*/*z* for: [C_20_H_15_ClN_6_S + H] calc. 407.0846 Found: 407.0835.

*10-[(1-(4-Cyanobenzyl)-1H-1,2,3-triazol-4-yl)methyl]-1,8-diazaphenothiazine* (**2e**). (135 mg, 69%) brown oil. ^1^H NMR (CDCl_3_) δ: 5.34 (s, 2H, CH_2_N_Th_), 5.56 (s, 2H. CH_2_N_Tr_), 6.80 (m, 1H, H_3_), 6.93 (m, 1H, H_6_), 7.21 (m, 1H, H_4_), 7.28 (m, 2H, H_Ph_), 7.53 (s, 1H, H_Tr_), 7.65 (m, 2H, H_Ph_), 7.96 (m, 1H, H_2_), 8.34 (m, 2H, H_7_ +H_9_). ^13^C NMR: 41.83, 53.33, 112.64, 114.95, 118.20, 118.68, 123.67, 125.31, 128.24, 129.05, 132.31, 132.86, 134.42, 135.22, 139.91, 144.82, 145.25, 145.41, 152.95. HRMS (EI) *m*/*z* for: [C_21_H_15_N_7_S + H] calc. 398.1189 Found: 398.1192.

*10-[(1-Phenylthiomethyl-1H-1,2,3-triazolo-4-ylo)methyl*]-*1,8-diazaphenothiazine* (**2f**). (150 mg, 75%) brown oil. ^1^H NMR (CDCl_3_) δ: 5.28 (s, 2H, CH_2_N_Th_), 5.57 (s, 2H. CH_2_N_Tr_), 6.83 (m, 1H, H_3_), 7.22 (m, 1H, H_6_), 7.28 (m, 6H, 5H_Ph_ + H_4_), 7.52 (s, 1H, H_Tr_), 7.96 (m, 1H, H_2_), 8.07 (m, 1H, H_7_), 8.23 (m, 1H, H_9_). ^13^C NMR: 41.69, 54.05, 114.39, 118.86, 120.80, 123.02, 128.85, 129.45, 129.53, 129.58, 131.55, 132.54, 132.79, 133.15, 133.75, 133.90, 134.45, 142.33, 144.45, 145.65, 152.54. HRMS (EI) *m*/*z* for: [C_20_H_16_N_6_S_2_ + H] calc. 405.0956 Found: 405.0974.

*10-[(1-Benzyl-1H-1,2,3-triazol-4-yl)-methyl]-2,7-diazaphenothiazine* (**3b**). (142 mg, 79%) yellow oil. ^1^H NMR (CDCl_3_) δ: 5.14 (s, 2H, CH_2_N_Th_), 5.52 (s, 2H, CH_2_N_Tr_), 6.64 (m, 1H), 6.99 (m, 1H), 7.21 (m, 2H), 7,35 (m, 3H), 7.36 (s, 1H, H_Tr_), 8.01 (m, 4H). ^13^C NMR: 44.49, 54.40, 109.72, 118.39, 121.95, 126.43, 126.98, 127.58, 128.92, 129.22, 129.33, 133.51, 134.23, 134.41, 141.09, 142.95, 144.83, 146.41. 149.45, 149.87. HRMS (EI) *m*/*z* for: [C_20_H_16_N_6_S + H] calc. 373.1235 Found373.1241.

*10-[(1-(4-Fluorobenzyl)-1H-1,2,3-triazol-4-yl)methyl]-2,7-diazaphenothiazine* (**3c**). (155 mg, 78%) beige oil. ^1^H NMR (CDCl_3_) δ: 5.13 (s, 2H, CH_2_N_Th_), 5.47 (s, 2H, CH_2_N_Tr_), 6.61 (d, *J = 5.4* Hz, 1H), 6.96 (d, *J = 5.4* Hz, 1H), 7.02 (m, 2H), 7.19 (m, 2H); 7.37 (s, 1H, H_Tr_), 7.94 (s, 1H), 8.03 (s, 1H), 8.07 (d, *J = 5.4* Hz, 1H), 8.12 (d, *J = 5.4* Hz, 1H). ^13^C NMR: 44.38, 53.63, 109.61, 116.16, 116.30, 121.57, 121.95, 121.86, 129.77, 129.83, 130.08, 130.10, 133.55, 135.40, 138.02, 138.09, 143.09, 144.83, 146.56. 149.57, 149.79. HRMS (EI) *m*/*z* for: [C_20_H_15_FN_6_S + H] calc. 391.1141 Found: 391.1140.

*10-[(1-(4-Chlorobenzyl)-1H-1,2,3-triazol-4-yl)methyl]-2,7-diazaphenothiazine* (**3d**). (154 mg, 82%) beige oil. ^1^H NMR (CDCl_3_) δ: 5.13 (s, 2H, CH_2_N_Th_); 5.49 (s, 2H, CH_2_N_Tr_), 6.62 (m, 1H), 6.97 (m, 1H), 7.17 (m, 2H), 7.35 (m, 2H), 7,39 (s, 1H, H_Tr_), 8,14 (m, 4H). ^13^C NMR: 44.49, 53.69, 109.73, 117.39, 121.57, 121.91, 129.26, 129.46, 132.64, 133.60, 135.05, 135.32, 138.09, 142.98, 144.90, 145.88, 149.01, 150.19. HRMS (EI) *m*/*z* for: [C_20_H_15_ClN_6_S + H] calc. 407.0846 Found: 407.0855.

*10-[(1-(4-Cyanobenzyl)-1H-1,2,3-triazol-4-yl)methyl]-2,7-diazaphenothiazine* (**3e**). (138 mg, 71%) brown oil. ^1^H NMR (CDCl_3_) δ: 5.11 (s, 2H, CH_2_N_Th_), 5.59 (s, 2H, CH_2_N_Tr_); 6.66 (m, 1H), 6.97 (m, 1H), 7.31 (m, 2H), 7.46 (s, 1H, H_Tr_), 8.04 (m, 1H), 8.17 (m, 3H). ^13^C NMR: 44.27, 53.64, 113.03, 118.39, 120.57, 121.37, 122.11, 122.30, 122.41, 132.87, 133.00, 137.60, 139.32, 143.33, 143.46, 144.12, 145.75, 146.20, 148.55, 149.19, 152.12. HRMS (EI) *m*/*z* for: [C_21_H_15_N_7_S + H] calc. 398.1189 Found: 398.1187.

*10-[(1-Phenylthiomethyl-1H-1,2,3-triazolo-4-ylo)methyl*]-*2,7-diazaphenothiazine* (**3f**). (155 mg, 79%) brown oil. ^1^H NMR (CDCl_3_) δ: 5.12 (s, 2H, CH_2_N_Th_), 5.59 (s, 2H, CH_2_N_Tr_), 6.59 (m, 1H), 7.04 (m, 1H), 7.24 (m, 2H), 7.29 (m. 3H), 7.35 (s, 1H, H_Tr_), 8.18 (m, 4H). ^13^C NMR: 44.36, 54.34, 109.81, 118.37, 121.63, 126.43, 126.98, 127.58, 128.54, 129.22, 129.33, 133.51, 134.23, 134.41, 135.31, 142.85, 144.90, 146.13. 149.13, 149.89. HRMS (EI) *m*/*z* for: [C_20_H_16_N_6_S_2_ + H] calc. 405.0956 Found: 405.0963.

*10-[(1-Benzyl-1H-1,2,3-triazol-4-yl)-methyl]-3,6-diazaphenothiazine* (**4b**). (144 mg, 80%) yellow oil. ^1^H NMR (CDCl_3_) δ: 5.07 (s, 2H, CH_2_N_Th_), 5.27 (s, 2H, CH_2_N_Tr_), 6.62 (m, 1H), 6.94 (m, 1H), 6.98 (m, 1H), 7.21 (m, 2H, H_Ph_), 7.36 (m, 3H, H_Ph_), 7.37 (s, 1H, H_Tr_), 8.03 (m, 1H), 8.19 (m, 2H). ^13^C NMR: 44.52, 54.45, 109.31, 121.40, 122.04, 122.12, 124.97, 126.39, 128.01, 128.87, 129.34, 130.44, 134.21, 137.68, 142.95, 144.03, 145.63, 145.96, 148.34, 149.25. HRMS (EI) *m*/*z* for: [C_20_H_16_N_6_S + H] calc. 373.1235 Found 373.1229.

*10-[(1-(4-Fluorobenzyl)-1H-1,2,3-triazol-4-yl)methyl]-3,6-diazaphenothiazine* (**4c**). (154 mg, 82%) beige oil. ^1^H NMR (CDCl_3_) δ: 5.03 (s, 2H, CH_2_N_Th_), 5.47 (s, 2H, CH_2_N_Tr_), 6.53 (m, 1H), 6.89 (m, 2H), 7.05 (m, 2H, H_Ph_), 7.19 (m, 2H, H_Ph_), 7.39 (s, 1H, H_Tr_), 7.95 (m, 1H), 8.05 (m, 1H), 8.10 (m, 1H). ^13^C NMR: 44.33, 53.63, 109.09, 115.84, 116.32, 118.68, 121.66, 122.04, 129.84, 130.14, 137.79, 143.19, 143.76, 145.70, 146.81, 148.76, 148.87, 149.09. HRMS (EI) *m*/*z* for: [C_20_H_15_FN_6_S + H] calc. 391.1141 Found: 391.1140.

*10-[(1-(4-Chlorobenzyl)-1H-1,2,3-triazol-4-yl)methyl]-3,6-diazaphenothiazine* (**4d**). (150 mg, 76%) beige oil. ^1^H NMR (CDCl_3_) δ: 5.07 (s, 2H, CH_2_N_Th_), 5.48 (s, 2H, CH_2_N_Tr_), 6.59 (m, 1H), 6.93 (m, 2H, H_Ph_), 7.16 (m, 2H, H_Ph_), 7.39 (s, 1H, H_Tr_), 7.95 (m, 1H), 8.02 (m,1H), 8.10 (m, 1H). ^13^C NMR: 44.21, 53.64, 109.19, 115.64, 116.22, 118.68, 121.86, 122.14, 129.94, 130.24, 137.89, 143.39, 143.96, 145.80, 146.85, 148.66, 148.97, 149.19. HRMS (EI) *m*/*z* for: [C_20_H_15_ClN_6_S + H] calc. 407.0846 Found: 407.0837.

*10-[(1-(4-Cyanobenzyl)-1H-1,2,3-triazol-4-yl)methyl]-3,6-diazaphenothiazine* (**4e**). (139 mg, 72%) brown oil. ^1^H NMR (CDCl_3_) δ: 5.11 (s, 2H, CH_2_N_Th_), 5.59 (s, 2H, CH_2_N_Tr_), 6.63 (m, 1H), 6.96 (m, 2H), 7.32 (m, 2H, H_Ph_), 7.48 (s, 1H, H_Tr_), 7.67 (m, 2H, H_Ph_), 8.05 (m, 1H), 8.14 (m, 2H). ^13^C NMR: 44.27, 53.65, 109.21, 113.05, 117.99, 121.36, 122.07, 122.11, 127.88, 128.01, 128.52, 132.83, 132.87, 133.02, 135.58, 137.60, 139.30, 143.47, 144.12, 148.34, 149.25. HRMS (EI) *m*/*z* for: [C_21_H_15_N_7_S + H] calc. 398.1189 Found: 398.1184.

*10-[(1-Phenylthiomethyl-1H-1,2,3-triazolo-4-ylo)methyl*]-*3,6-diazaphenothiazine* (**4f**). (153 mg, 78%) brown oil. ^1^H NMR (CDCl_3_) δ: 5.05 (s, 2H, CH_2_N_Th_), 5.62 (s, 2H, CH_2_N_Tr_), 6.55 (m, 1H), 6.93 (m, 1H), 6.97 (m, 1H), 7.24 (m, 5H), 7.33 (s, 1H, H_Tr_), 8.07 (m, 1H), 8.15 (m, 2H). ^13^C NMR: 44.45, 54.26, 109.01, 121.43, 121.72, 122.21, 129.08, 129.58, 131.21, 132.52, 137.37, 142.66, 144.33, 145.01, 145.38, 147.62, 149.76. HRMS (EI) *m*/*z* for: [C_20_H_16_N_6_S_2_ + H] calc. 405.0956 Found: 405.0955.

### 3.2. Anticancer Effects In Vitro

#### 3.2.1. Cell Culture

All dipyridothiazines with 1,2,3-triazole substituents (**1**–**4)b**–**f** were evaluated for their anticancer activity using four cultured cell lines: SNB-19 (human glioblastoma, DSMZ—German Collection of Microorganisms and Cell Cultures, Braunschweig, Germany), Caco-2 (human colon adenocarcinoma, DSMZ—German Collection of Microorganisms and Cell Cultures, Braunschweig, Germany), A549 (human lung carcinoma, ATCC, Manassas, VA, USA) and MDA-MB231 (human breast adenocarcinoma, ATCC, Manassas, VA, USA), and NHDF (human dermal fibroblast cell line, ATCC, Manassas, VA, USA. The cultured cells were kept at 37 °C and 5% CO_2_. The cells were seeded (1 × 10^4^ cells/well/100 µL DMEM supplemented with 10% FCS and streptomycin and penicillin) using 96-well plates (Corning). The cells were counted in a hemocytometer (Burker’s chamber) using a phase contrast Olympus IX50 microscope equipped with Sony SSC-DC58 AP camera and Olympus DP10 digital camera.

#### 3.2.2. Cell Proliferation and Viability

Antiproliferative effect of the obtained compounds on the cancer cells was determined using the cell proliferation reagent WST-1 assay (Roche Diagnostics, Mannheim, Germany). This assay is based on the viable cell’s ability to cleave the bright red-colored tetrazolium salt (2-(4-iodophenyl)-3-(4-nitrophenyl)-5-(2,4-disulfophenyl)-2H-tetrazolium, monosodium salt) to dark red soluble formazan by cellular enzymes. An increase in the amount of formazan dye formed correlates to the number of metabolically active cells in the culture. The formazan dye produced by metabolically active cells is quantified by a scanning ELISA reader by measuring the absorbance of the dye solution at appropriate wavelengths. The examined cells were exposed to the tested compounds for 72 h at various concentrations between 0.1 μg/mL and 100 μg/mL (prepared initially at concentration of 1 mg/mL in DMSO). The cells were incubated with WST-1 (10 μL) for 1 h and the absorbance of the samples against a background control was measured at 450 nm using a microplate reader with a reference wavelength at 600 nm. The results are expressed as means of at least two independent experiments performed in triplicate. The antiproliferative activity of the tested compound was compared to cisplatin. The IC_50_ values (a concentration of a compound that is required for 50% inhibition) were calculated from the dose-response relationship with respect to control.

#### 3.2.3. The RT-qPCR Method

Genes trancriptional activity (*H3, TP53, CDKN1A, BCL-2*, and *BAX*) was evaluated by real time RT-qPCR with OPTICON TM DNA Engine (MJ Research, Watertown, MA, USA) and QuantTect^®^ SYBR^®^ Green RT-PCR Kit (Quiagen, Valencia, CA). Cells were exposed to compound 4 at a 0.5 µg/mL concentration for 24 h. The RNA extraction was made by using Quick-RNA™ Kit MiniPrep (ZYMO RESEARCH). Total RNA integrity was analysed in 1.2% agarose electrophoresis with added ethidium bromide. The quantity and purity of extracted total RNA were determined by using spectrophotometric analysis with HP845 (Hewlett Packard, Waldbronn, Germany) spectrophotometer. The statistical analysis was performed using the Statistica 8.0 software. All values were expressed as means ± SE.

## 4. Conclusions

We report here efficient synthesis of a new isomeric series of novel 1,2,3-triazole-dipyridothiazine hybrids in the 1,3-dipolar cycloadditon reactions. The structure of these new compounds was identified using advanced two-dimensional ^1^H and ^13^C NMR (COSY, ROESY) spectra. Most of the obtained derivatives of diazaphenothiazines exhibited significant anti-proliferative activities against the human glioblastoma SNB-19, colorectal carcinoma Caco-2, breast cancer MDA-MB-231 and lung cancer A549 cell lines with the IC_50_ values < 1 μM and were more potent than cisplatin. The most promising compound **1d** was used for gene expression analysis by the RT-qPCR method. The expression of *H3, TP53, CDKN1A, BCL-2,* and *BAX* genes revealed that this compound inhibited the proliferation in all cells (*H3*) and activated mitochondrial events of apoptosis (*BCL-2/BAX*) in MDA-MB231 cancer cell line. Further in vitro and in vivo studies are required to determine the potential pharmacological use of these dipyrido-1,2,3-triazole hybrids in anticancer therapy.

## Figures and Tables

**Table 1:** The ^1^H NMR chemical shifts and proton-proton correlations (ROESY and COSY experiments) for compound **1b**.

^1^H NMR	ROESY	COSY
5.22 CH_2_N_Th_	5.22/7.46	5.22/7.61
5.49 CH_2_N_Tr_	5.49/7.23/7.61	5.49/7.23/7.61
6.76 H_3_	6.76/7.23/7.92	6.76/7.23/7.92
6.93 H_8_	6.93/7.46	6.93/7.46/7.92
7.23 *o*-H_Ph_, 1H_4_	7.23/6.76/7.35	7.23/6.76/7.35/7.92
7.35 *m,p*-H_Ph_	7.35/7.23	7.35/7.23
7.46 H_9_	7.61/5.22	7.46/6.93/7.92
7.61 H_Tr_	7.61/5.49/7.23	7.61/5.22
7.92 H_7_ + H_2_	7.92/6.76/6.93	7.92/6.76/6.93/7.23/7.46

**Table 2:** Anticancer activities IC_50_ (μM) of 10-substituted 3,6-diazaphenothiazines (**1b**–**4f**) against glioblastoma SNB-19, colorectal carcinoma Caco-2, lung cancer A549, breast cancer MDA-MB231 cells and normal human fibroblasts NHDF.

No	Anticancer Activity IC_50_ (μM)				
	SNB-19	Caco-2	A549	MDA-MB231	NHDF
**1b**	69.15+/−3.14	19.92+/-2.71	>100	13.66+/−1.16	>100
**1c**	5.72+/−1.44	8.10+/−0.92	29.12+/−2.59	7.82+/−1.32	>100
**1d**	4.66+/−1.16	0.25+/−0.02	0.25+/−0.01	0.51+/−0.09	>100
**1e**	9.65+/−1.99	79.65+/−4.12	1.79+/−0.62	25.79+/−1.32	>100
**1f**	>100	>100	>100	68.68+/−5.12	>100
**2b**	41.91+/−3.15	>100	77.44+/−3.22	56.34+/−5.72	>100
**2c**	55.23+/−5.78	43.61+/−6.52	60.23+/−2.77	9.21+/−3.01	>100
**2d**	59.87+/−9.99	11.42+/−2.13	1.82+/−0.71	4.71+/−0.22	42.95+/−5.11
**2e**	10.62+/−1.11	34.38+/−2.72	63.75+/−2.78	12.04+/−2.21	>100
**2f**	56.34+/−5.72	32.55+/−8.21	46.80+/−2.73	12.04+/−6.11	>100
**3b**	2.04+/−0.21	0.26+/−0.01	0.26+/−0.11	0.77+/−0.10	22.66+/−1.32
**3c**	57.92+/−4.96	29.87+/−5.21	27.23+/−2.16	25.66+/−4.11	70.61+/−12.21
**3d**	31.31+/−10.99	33.07+/−9.18	17.91+/−1.11	4.26+/−0.15	>100
**3e**	26.80+/−1.72	10.02+/−0.99	30.07+/−3.21	3.50+/−0.35	49.47+/−1.11
**3f**	32.58+/−2.77	14.54+/−1.22	0.65+/−0.10	0.64+/−0.10	52.90+/−3.21
**4b**	20.80+/−1.96	57.90+/−2.15	60.62+/−3.52	7.87+/−1.11	>100
**4c**	6.25+/−0.62	0.25+/−0.02	0.25+/−0.01	1.97+/−0.13	2.85+/−0.34
**4d**	14.87+/−1.70	42.06+/−2.99	53.99+/−10.72	13.44+/−1.22	>100
**4e**	53.62+/−3.72	74.28+/−4.72	42.69+/−13.72	38.77+/−2.12	>100
**4f**	44.83+/−13.62	67.52+/−11.87	>100	44.33+/−11.79	>100
***Cisplatin***	3.73+/−0.62	10.53+/−1.52	0.60+/−0.11	3.13+/−0.24	63.87+/−1.32

**Table 3:** The influence of compound **1d** on expression of genes encoding *H3, TP53, CDKN1A, BCL-2,* and *BAX* in glioblastoma SNB-19, colorectal carcinoma Caco-2, lung cancer A549, and breast cancer MDA-MB231 cells.

gene		SNB-19	Caco-2	A549	MDA-MB231
		Number of mRNA Copies/μg Total RNA			
*H3*	control	143+/−41	11+/−3	23+/-13	2+/−1
	**1d**	52+/−11	45+/−3	18+/−5	8+/−6
*TP53*	control	198+/−52	65+/−24	49+/−17	56+/−16
	**1d**	128+/−24	193+/−16	54+/−19	46+/−20
*CDKN1A*	control	698+/−34	266+/−49	1012+/−143	710+/−486
	**1d**	308+/−51	260+/−21	969+/−137	577+/−308
*BCL-2*	control	70604+/−7852	673718+/−52998	22485+/−4794	207802+/−15572
	**1d**	17497+/−4607	723708+/−217134	15631+/−2296	16495+/−4607
*BAX*	control	995+/−98	1062+/−141	1181+/−132	835+/−63
	**1d**	220+/−18	969+/−264	835+/−231	221+/−16
*BCL-2/BAX*	control	71	634	19	249
	**1d**	80	743	18	80

## Supplementary Materials

The supplementary materials are available online.
