# Supplementary material for: Design, Synthesis, and Structural Characterization of Novel Diazaphenothiazines with 1,2,3-Triazole Substituents as Promising Antiproliferative Agents
Source: Molecules. 2019 Nov 30;24(23):4388. doi: 10.3390/molecules24234388 (PMC6930555; doi:10.3390/molecules24234388)
Supplement: Supplementary file 1 [file molecules-24-04388-s001.pdf]

Supplementary Material

Design, synthesis, structural characterization of novel diazaphenothiazines with 1,2,3-triazole substituents as promising anticancer agents

Beata Morak-Młodawska[1]*, Krystian Pluta[1], Małgorzata Latocha[2], Małgorzata Jeleń[1], Dariusz Kuśmierz[2]

[1]The Medical University of Silesia, Faculty of Pharmaceutical Sciences, Department of Organic Chemistry, Jagiellońska 4, 41-200 Sosnowiec, Poland,

[2]The Medical University of Silesia, Faculty of Pharmaceutical Sciences, Department of Cell Biology, Jedności 8, 41-200 Sosnowiec, Poland.

Content 1

1. ^{1}H NMR of 10-[(1-Benzyl-1H-1,2,3-triazol-4-yl)-methyl]-1,6-diazaphenothiazine (**1d**) 2
2. ^{1}H NMR of the compound (**9**) – aromatic part 3
3. COSY NMR of 10-[(1-Benzyl-1H-1,2,3-triazol-4-yl)-methyl]-1,6-diazaphenothiazine (**1d**) 4
4. ROESY NMR of 10-[(1-Benzyl-1H-1,2,3-triazol-4-yl)-methyl]-1,6-diazaphenothiazine (**1d**) 5
5. ROESY NMR of 10-[(1-Benzyl-1H-1,2,3-triazol-4-yl)-methyl]-1,6-diazaphenothiazine (**1d**) – aromatic part 6
6. ^{13}C NMR of 10-[(1-Benzyl-1H-1,2,3-triazol-4-yl)-methyl]-1,6-diazaphenothiazine (**1d**) 7
7. ^{13}C NMR of 10-[(1-Benzyl-1H-1,2,3-triazol-4-yl)-methyl]-1,6-diazaphenothiazine (**1d**) – aromatic part 8
8. 8. HR MS of 10-[(1-Benzyl-1H-1,2,3-triazol-4-yl)-methyl]-1,6-diazaphenothiazine (**1d**) 9

1. ^{1}H NMR of 10-[(1-Benzyl-1H-1,2,3-triazol-4-yl)-methyl]-1,6-diazaphenothiazine (**1d**)

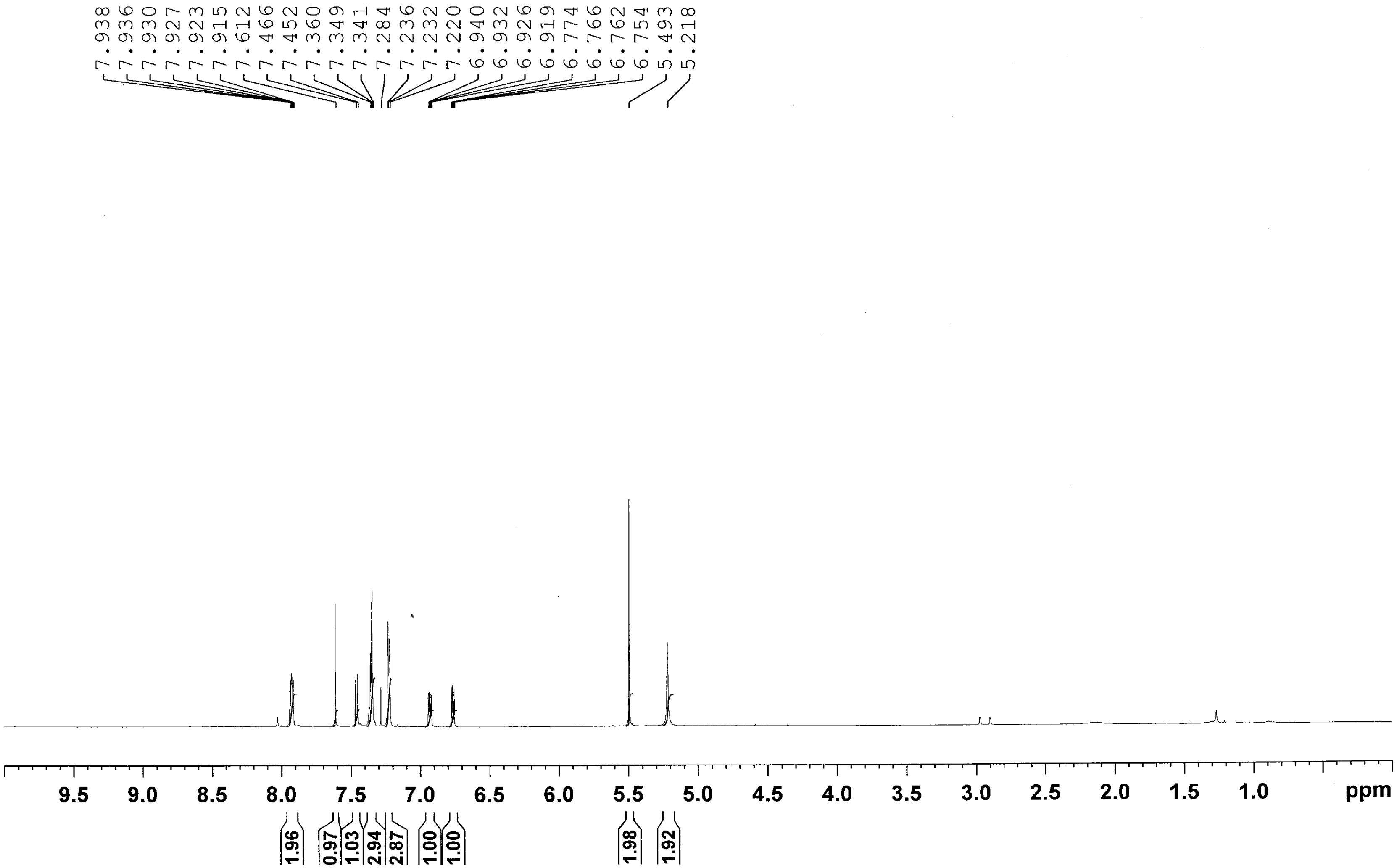

2. ^{1}H NMR of the compound (**1d**) – aromatic part

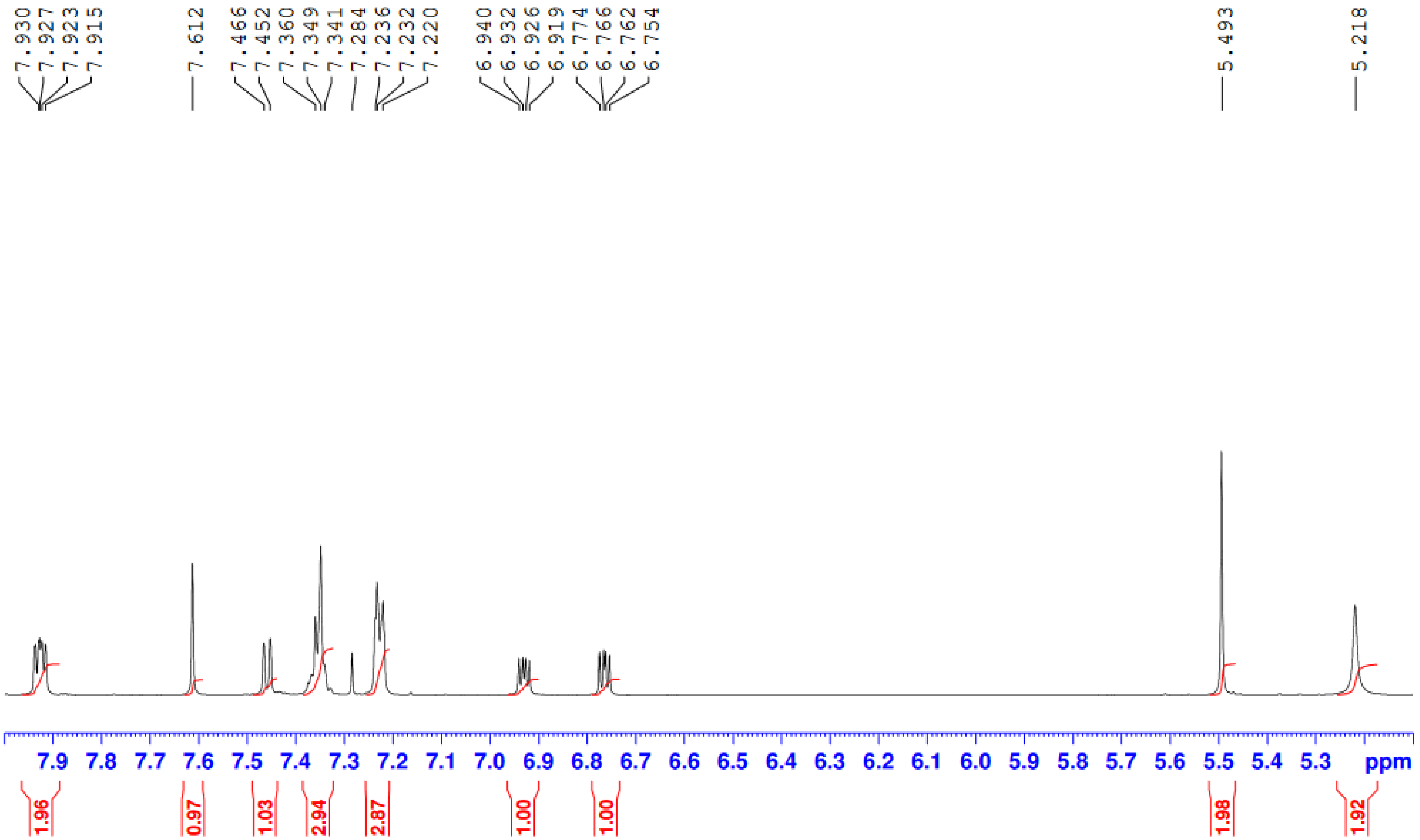

3. COSY NMR of 10-[(1-Benzyl-1H-1,2,3-triazol-4-yl)-methyl]-1,6-diazaphenothiazine (**1d**)

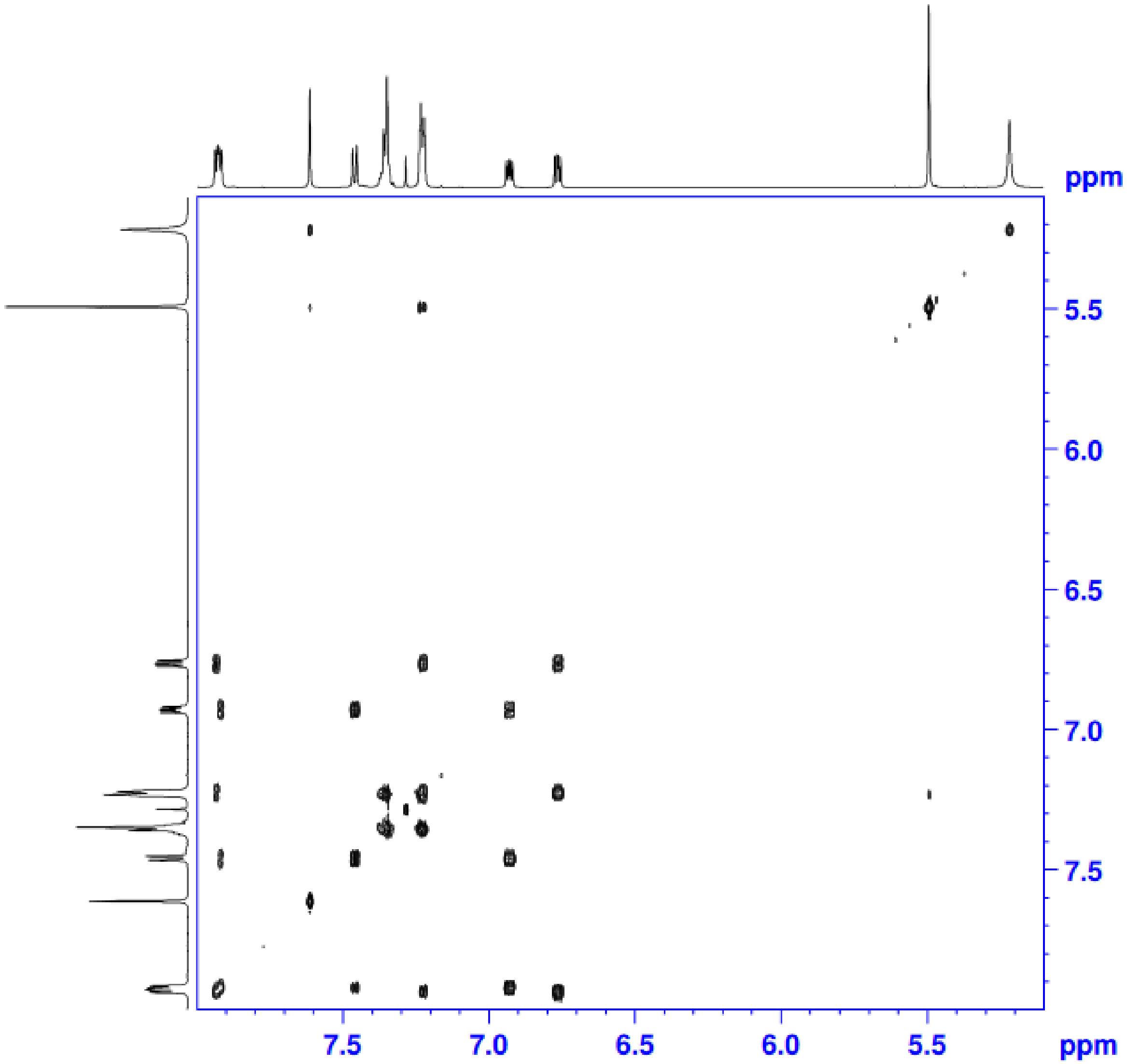

4. ROESY NMR of 10-[(1-Benzyl-1H-1,2,3-triazol-4-yl)-methyl]-1,6-diazaphenothiazine (**1d**)

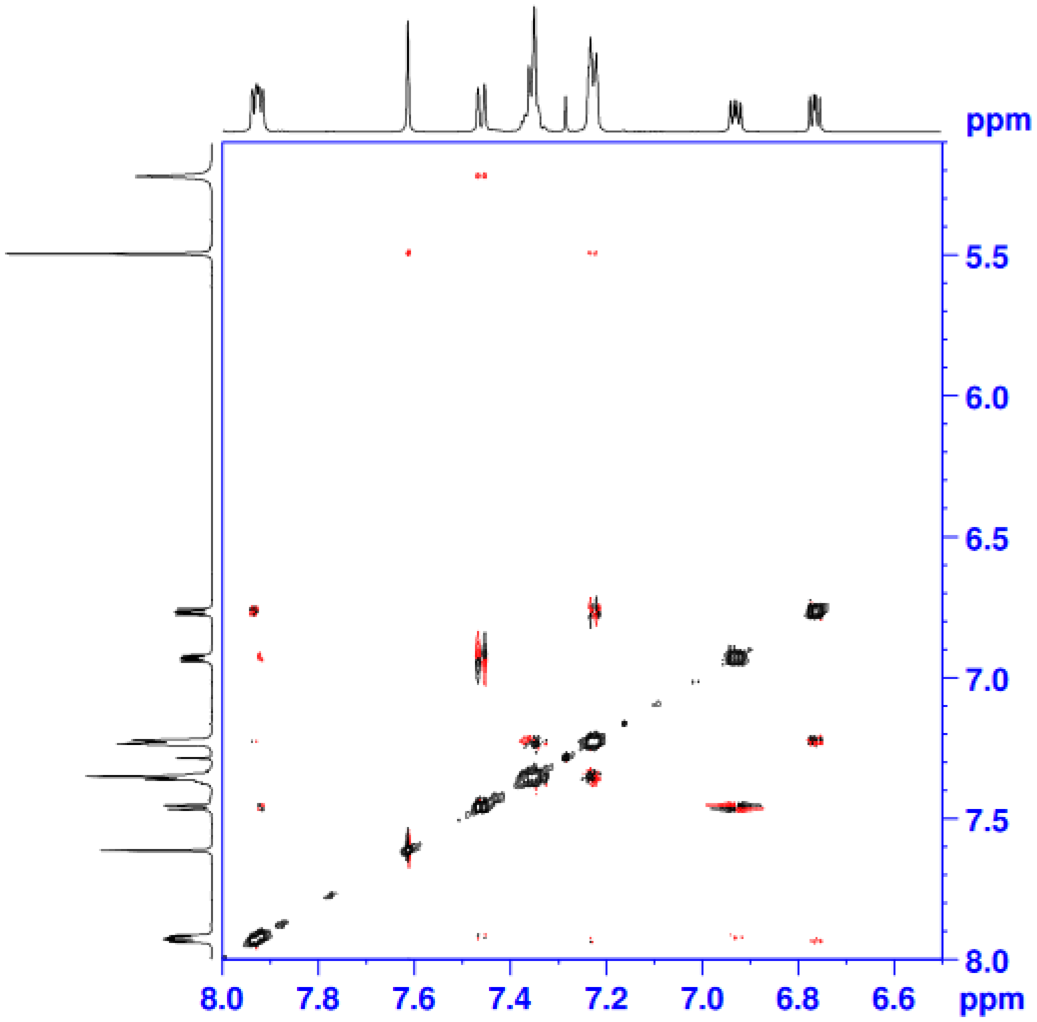

5. ROESY NMR of 10-[(1-Benzyl-1H-1,2,3-triazol-4-yl)-methyl]-1,6-diazaphenothiazine (**1d**) – aromatic part

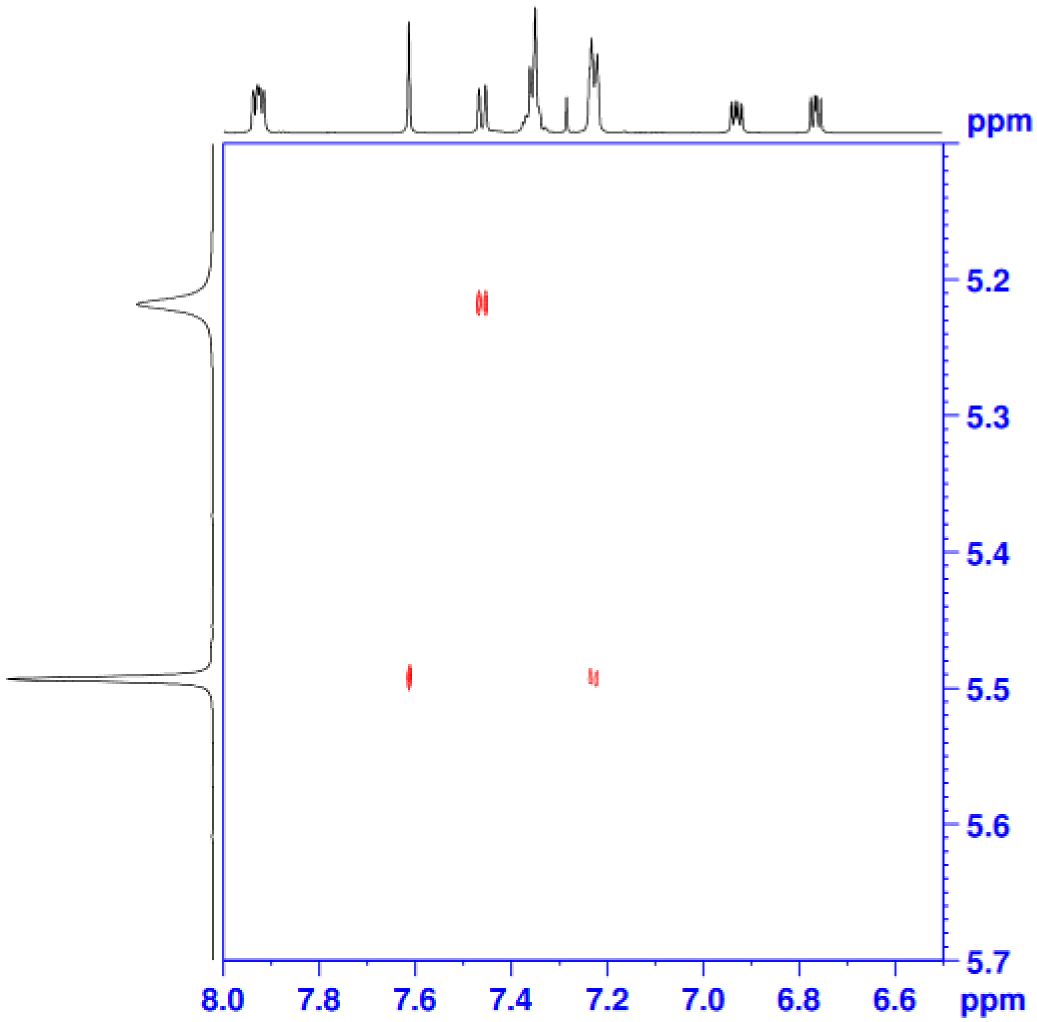

6. ^{13}C NMR of 10-[(1-Benzyl-1H-1,2,3-triazol-4-yl)-methyl]-1,6-diazaphenothiazine (**1d**)

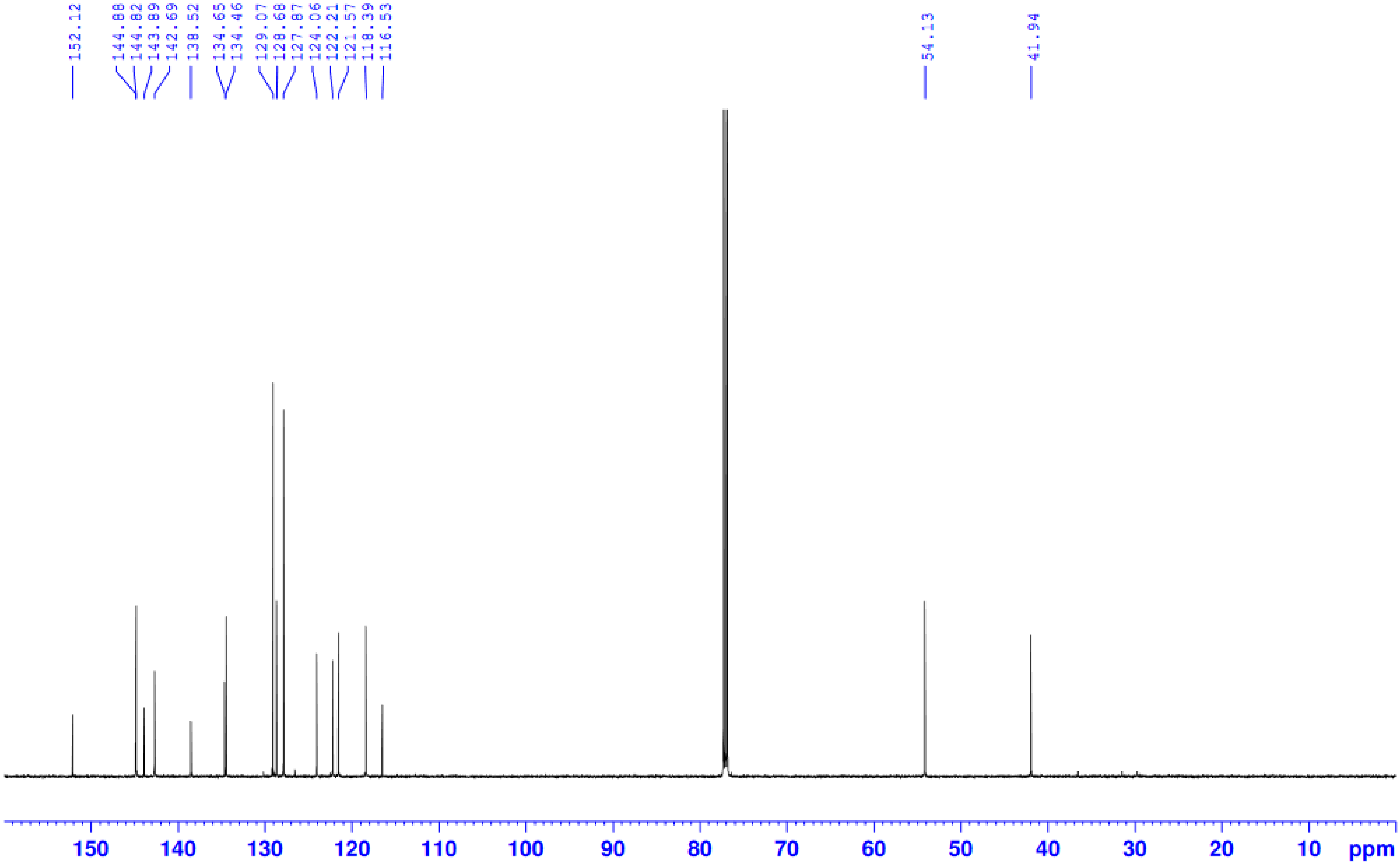

7. ^{13}C NMR of 10-[(1-Benzyl-1H-1,2,3-triazol-4-yl)-methyl]-1,6-diazaphenothiazine (**1d**) – aromatic part

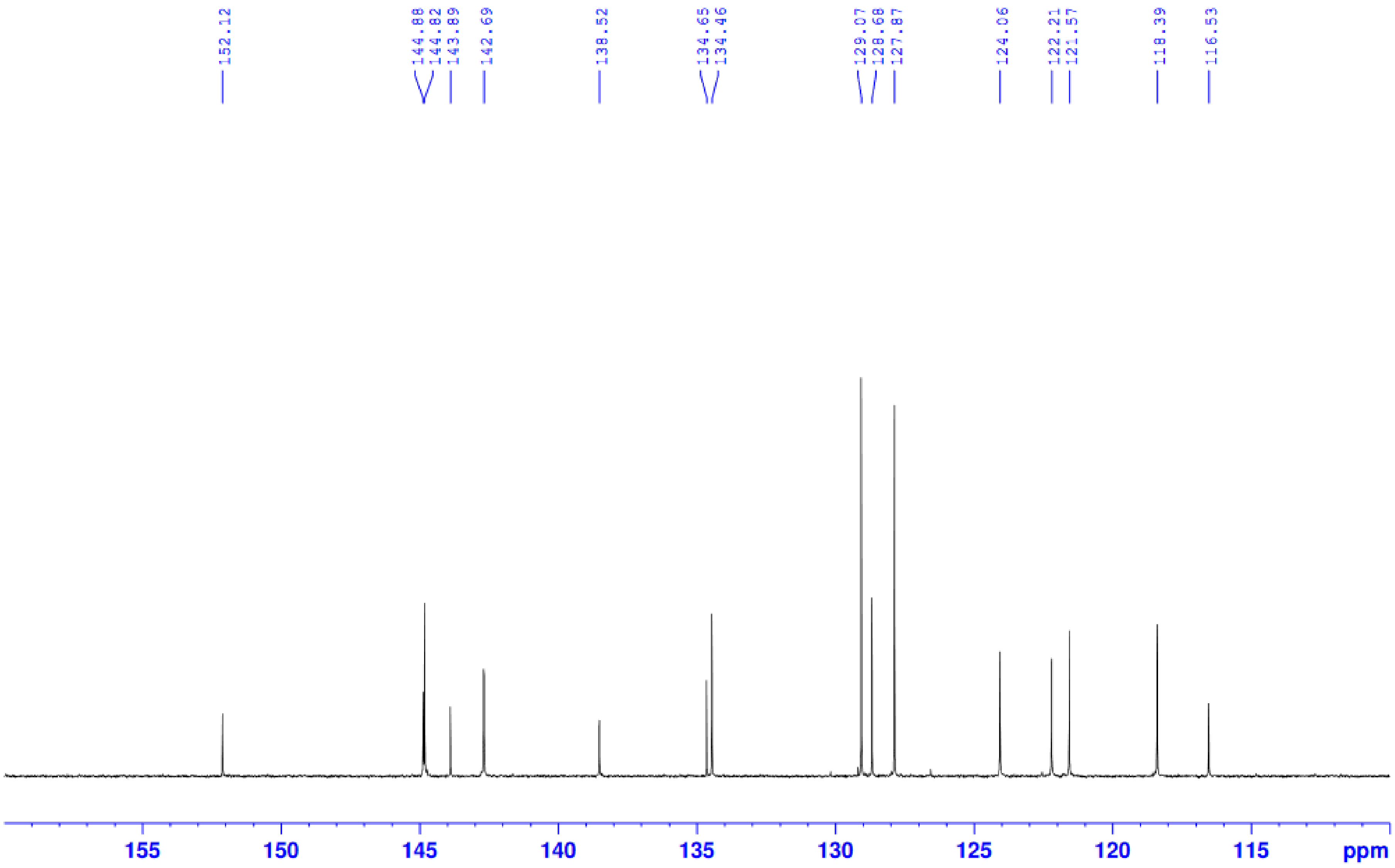

8. HR MS of 10-[(1-Benzyl-1H-1,2,3-triazol-4-yl)-methyl]-1,6-diazaphenothiazine (**1d**)

Compound Spectrum List Report

Analysis Info

Analysis Name	D:\Data\BA78.d	Acquisition Date	11/14/2017 12:55:17 PM	
Method	low_mass.m	Operator	KM	
Sample Name	TM Low concentration	Instrument	impact II	1825265.10082
Comment				

Acquisition Parameter

Source Type	ESI	Ion Polarity	Positive	Set Nebulizer	0.3 Bar
Focus	Active	Set Capillary	4000 V	Set Dry Heater	240 °C
Scan Begin	100 m/z	Set End Plate Offset	-500 V	Set Dry Gas	4.0 l/min
Scan End	1000 m/z	Set Charging Voltage	2000 V	Set Divert Valve	Source
		Set Corona	0 nA	Set APCI Heater	0 °C

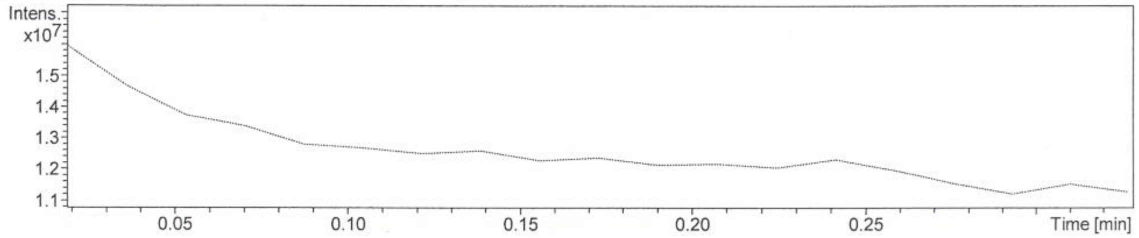

+MS, 0.0-0.3min #2-18

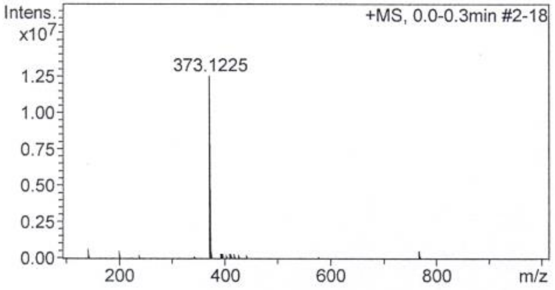

#	m/z	Res.	S/N	I	I %	FWHM
1	373.1225	43747	113088.6	12532882	100.0	0.0085
